# Preventing Transmission of Lethal Disease: Removal Behaviour of *Lasius fuliginosus* (Hymenoptera: Formicidae) Towards Fungus Contaminated Aphids

**DOI:** 10.3390/insects12020099

**Published:** 2021-01-24

**Authors:** Tatiana Novgorodova

**Affiliations:** Institute of Systematics and Ecology of Animals, Siberian Branch of the Russian Academy of Sciences, Frunze str. 11, Novosibirsk 630091, Russia; tanovg@yandex.ru

**Keywords:** social immunity, ants, trophobiosis, entomopathogenic fungi, prophylactic mechanism, quarantining behaviour, *Beauveria bassiana*, *Chaitophorus populeti*

## Abstract

**Simple Summary:**

To decrease the risk of infection transmission, ants are known to use a number of defensive mechanisms. One of them is the removal of conidia-contaminated aphids and sporulating cadavers, which is aimed at limiting contacts with potentially dangerous entities. This quarantining behaviour is helpful in limiting transmission of disease by the aphid milkers, both to the ant colony and among aphids, which are their main suppliers of carbohydrate food. The spread of this behaviour among ants is still scarcely studied. Among seven ant species studied, active usage of quarantining behaviour was found only in Formica ants. The behaviour of *Lasius fuliginosus* aphid milkers towards *Chaitophorus populeti* aphids covered with conidia of *Beauveria bassiana* was studied in the field. Most aggressive milkers quickly detected and removed conidia-contaminated aphids from the plant, carrying them down and placing them some distance away from the experimental aspen trees. In general, active usage of quarantining behaviour towards fungus contaminated aphids was found to be not limited to the genus *Formica*, but typical of *L. fuliginosus* as well. Removal of most fungus-contaminated aphids from the plant enables these ants to reduce the risk of infection transmission among aphids, as well as among ants.

**Abstract:**

The ability of ants to detect and remove conidia-contaminated aphids, aimed at limiting contacts with potentially dangerous entities, is an effective antifungal mechanism to prevent the spread of infection among both their nestmates and aphids, their main suppliers of carbohydrates. However, the spread and the scale of this quarantining behaviour among ants are still scarcely studied. Among seven ant species studied, active usage of quarantining behaviour was found only in Formica ants. The behaviour of *Lasius fuliginosus* (Latreille) aphid milkers towards *Chaitophorus populeti* (Panzer) aphids covered with conidia of *Beauveria bassiana* (Balsamo-Crivelli) Vuillemin was studied in the field. Most aggressive milkers quickly detected and removed conidia-contaminated aphids from the plant, carrying them down and placing them some distance away from the experimental aspen trees. In general, active usage of quarantining behaviour towards conidia-contaminated aphids was found to be not limited to the genus *Formica*, but typical of *L. fuliginosus* as well. The response of milkers of *L. fuliginosus* and *Formica* s. str. ants to living aphids covered with conidia is quite similar. Removal of most fungus-contaminated aphids from the plant enables these ants to reduce the risk of infection transmission among both their nestmates and aphids.

## 1. Introduction

Eusocial insects are known to be highly successful due to the complex social organization of their colonies based on clear divisions of labour [1,2,3]. However, besides various benefits, group living bears some significant costs. Densely populated colonies of eusocial insects are a perfect target for rapid transmission of pathogenic microorganisms, which could potentially be a serious threat to these insects [4,5,6]. To withstand the increased risk of infection transmission, eusocial insects developed a number of collective anti-parasite defensive practices including both prophylactic and activated on demand mechanisms, termed ‘social immunity’ [7,8,9]. In ants, this collective defence includes a broad range of behaviours aimed at avoidance, control or disposal of pathogenic microorganisms: chemical disinfection through the application of poison secretion produced by ants [10,11] and tree resin [12,13], (allo-)grooming [14,15,16], corpse removal and burying infected nestmates [17,18,19,20], prophylactic wound care [21] and even becoming unsociable when infected [22,23].

Recent studies on ants have shown that the anti-fungal defences of these insects are not limited to ‘housekeeping’ and interactions between nestmates, but also may involve symbionts playing an important role in their life. The prosperity of ant colonies is known to be highly affected by the well-being of their trophobiont partners, including aphids, which are their main suppliers of energy-rich carbohydrate food [24,25,26]. Due to aphids living in densely populated colonies, as well as their active migrations within and between host plants, aphids can serve as vectors of fungal diseases [27,28,29]. Close ant-aphid interactions require special measures to prevent the spread of infection among both trophobionts to avoid their loss, and the loss of ants. Besides protection of aphids against various aphidophagous insects [30,31,32], ants have been found to detect and remove fungus contaminated aphids [33,34]. Removal behaviour towards both sporulating cadavers and living aphids covered with conidia is aimed at limiting contacts (isolation) with potentially dangerous entities. In addition to detecting and eliminating fungus contaminated aphids or their sporulating corpses from the plant, removal behaviour of ants may also include killing fungus-exposed aphids, their disinfection using poisonous secretion, as well as, presumably, their burial. This hygienic behaviour enables ants to prevent or significantly decrease the possibility of transmission of dangerous infections, so it is referred to as quarantining behaviour [33,34]. Removal of fungus contaminated aphids seems to be more effective than grooming, which can be useless in preventing infection transmission, if concentration of fungus conidia is high or it is too late for (allo-)grooming [16]. Removal behaviour was demonstrated by ants towards aphids contaminated with conidia of both obligate, aphid-specific fungal pathogen *Pandora neoaphidis* (Remaudie and Hennebert) Humber (Entomophthorales) [33] and the generalist fungus *Beauveria bassiana* (Balsamo-Crivelli) Vuillemin (Hypocreales) [34]. In general, on the one hand, quarantining behaviour is helpful in limiting transmission of disease to the ant colony by the aphid milkers transporting collected aphid honeydew to the nest. On the other hand, this decreases the risk of milkers being a vector of infection transmission among their trophobiont partners, which can lead to a decrease or loss of this important carbohydrate resource. Most importantly, removal of living aphids covered with conidia prevents the appearance of sporulating corpses of aphids on the host-plant, thereby highly decreasing the risk of infection transmission in total. Thereby ants defend trophobionts, as well as themselves. 

Until recently, behaviour of aphid milkers towards fungus contaminated aphids was only studied in a limited number of species. Among seven ant species studied so far, only ants of the genus *Formica* were found to use removal behaviour quite actively [33,34,35]. As for *Lasius niger* (Linnaeus, 1758), the only ant of the genus *Lasius* studied so far, aphid milkers of this ant species tend to demonstrate non-aggressive reactions towards aphids covered with conidia (neutral reaction, antennation, honeydew collection, grooming) without making any difference between ‘familiar’ aphids and ‘newcomers’ covered with conidia [34]. However, the hypothesis of this paper is that this antifungal mechanism is unlikely to be limited to the genus *Formica* and should be more widespread among ants. Besides, it is likely to be quite diverse within the same ant genus, including *Lasius*, because ant behaviour is known to be highly affected by the ant colony size [36]. Ant colonies that were of larger size were found to demonstrate a higher complexity of territorial and foraging behaviour [37,38,39]. 

In the genus *Lasius*, *Lasius fuliginosus* (Latreille, 1798) stands out from other ants because of its larger size and the more complex territorial organization of its colonies, which are similar to those of obligate dominant ants of *Formica* s. str. [2,40,41]. In addition, the effectiveness of protection of aphid colonies against aphidophages in *L. fuliginosus* tends to be higher than in *L. niger* and closer to *Formica* s. str. ants [32]. Based on these data, it is hypothesized that *L. fuliginosus* has complex defensive behaviours, including the ability to detect and remove conidia-contaminated aphids as *Formica* s. str. ants tend to do. In order to test this hypothesis, the reaction of *L. fuliginosus* milkers towards aphids covered with conidia of a generalist entomophathogenic fungus *Beauveria bassiana* was investigated. The behaviour of *L. fuliginosus* towards fungus-contaminated aphids and the ability of this ant to prevent infection transmission among its symbiont partners had not been studied before. This study was focused on two main issues: (i) whether aphid milkers of *L. fuliginosus* can detect and remove living aphids contaminated with conidia; (ii) whether there are any differences between *L. fuliginosus* and *Formica* s. str. ants in their response to aphids covered with conidia of *B. bassiana*.

## 2. Materials and Methods 

### 2.1. Study Area and Species

Experimental investigations of the reaction of *Lasius fuliginosus* towards living aphids externally contaminated with conidia of entomopathogenic fungus *Beauveria bassiana* were carried out in July 2018 and 2019 in two districts of the Novosibirsk Region (Western Siberia, Russia). Two colonies of *L. fuliginosus* were chosen as a model in aspen-birch groves in Karasuk District (53°44′ N 78°02′ E; 2018), and one more colony was studied in aspen-birch-pine forest near Novosibirsk (54°83’N 83°12’E; 2019). The ants were identified using Radchenko [42,43] and Czechowski et al. [40]. The aphids were studied on microscope slides prepared using Faure–Berlese fluid and identified using Blackman and Eastop [44]. The materials relating to the ants and the aphids were deposited at the Institute of Systematics and Ecology of Animals SB RAS (Novosibirsk, Russia).

*Lasius fuliginosus* is widely distributed in Europe and Asia, as far east as Altai, inhabiting forest biotopes mainly in forest-steppe and in the south of forest zone [43]. This ant constructs carton nests at the base of old trees, usually lives in populous polydomous colonies (up to 500,000 workers) and possesses quite large protected feeding territories (up to 0.3 ha) with well-developed systems of tunnels and above ground trails [41]. In multispecies ant communities, *L. fuliginosus* plays a role of obligate dominant, along with ants of *Formica* s. str. [41]. The diet of this ant includes various small arthropods and the honeydew of sap-feeding insects [41]. Functional differentiation among aphid milkers is not typical of this ant; *L. fuliginosus* applies the simplest honeydew collecting strategies through unspecialized foragers in ‘protected’ or ‘unprotected’ aphid colonies [45]. Nevertheless, the degree of protection *L. fuliginosus* provides aphids against their natural enemies is quite high and similar to that provided by *Formica* s. str. ants [32].

**Figure 1:**
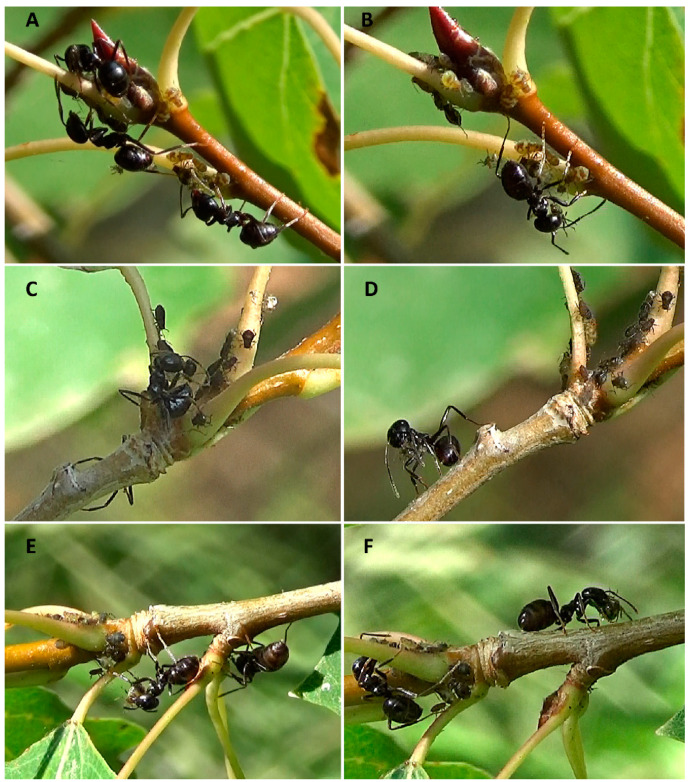
Aphid milkers of *Lasius fuliginosus* in experimental aphid colonies of *Chaitophorus populeti populeti* on young aspen trees (*Populus tremula*). (**A**) Antennation of uncontaminated aphid from the control group. (**B**–**F**) Removal of aphids externally contaminated with conidia: grabbing an aphid (**B**,**E**), bending abdomen round as if ant were spraying poison to the grabbed aphid (**C**), carrying aphid down the plant (**D**,**F**).

Ant behaviour was observed on young aspen trees (*Populus tremula* Linnaeus, 1753, Salicaceae) naturally infested with aphids of the obligate myrmecophilous species *Chaitophorus populeti populeti* (Panzer, 1801) (Hemiptera: Aphididae) (Figure 1). This aphid is widespread throughout the Palaearctic region, tends to form colonies on young shoots and terminal leaf petioles of various *Populus* spp. and is usually attended by ants [44]. Ten aspen trees of about 2.0–3.5 m height located at a distance of about 2.5–12.5 m were chosen for each ant colony explored. To avoid testing the same ant foragers twice, either one or two model aphid colonies located at maximum possible distance from each other (i.e., on different branches on opposite sides of the plant) were chosen on each tree. Earlier *Lasius fuliginosus* was found to be able to detect and attack ‘unfamiliar’ aphid individuals visited by milkers from other conspecific colonies [46]. To avoid such an effect, experimental aphids of *C. populeti* were collected directly before testing from the conspecific aphid colonies located on the other branches of the experimental aspen tree and tended by foragers from the same ant colony. 

As for *Beauveria bassiana* s.l., it is known to be a widespread entomophathogenic fungus attacking an extremely broad range of hosts, including aphids and ants [47,48,49,50]. In this study, *Beauveria bassiana*, isolate Sar-31 (the collection of entomopathogenic fungal cultures of the Institute of Systematics and Ecology of Animals SB RAS, Novosibirsk, Russia), isolated from an egg pod of the locust *Calliptamus italicus* (Linnaeus, 1758) (Orthoptera) in the south of Western Siberia in 2001 was used to contaminate aphids. The conidia of *B. bassiana* (isolate Sar-31) were grown in twice-autoclaved millet [51] and then sifted through a soil sieve and stored at 4 °C. The quantity of conidia was 2 × 10^7^ per mg of conidial mass. The conidia titre was determined using a Neubauer hemocytometer (conidia were suspended in sterile 0.03% (*v*/*v*) aqueous Tween 20). The level of conidia germination on Sabouraud dextrose agar was 100%.

### 2.2. Experimental Design

The ability of *Lasius fuliginosus* milkers to detect and remove aphids covered with conidia was investigated according to Novgorodova and Kryukov [34]. One half of experimental aphids were treated with *Beauveria bassiana* (hereafter referred to as fungus or conidia-contaminated) using a standard method of dipping insect in a suspension of fungus conidia [52,53], which was preliminary adapted for aphids. Each of these individuals was submerged for 2–3 s in the conidia suspension in distilled water (2 × 10^7^ conidia ml^−1^). During our preliminary investigations, aphids were found to be quite sensitive to Tween. So, to avoid the negative effect of Tween, conidia suspension in water was used. In order to minimize the lump formation problem, the water suspension of the conidia was actively shaken each time immediately before treatment of the experimental aphid. The control group consisted of aphids treated with distilled water. Every experimental aphid was treated just before testing. Only actively moving aphids behaving as usual after the treatment were used in the tests. Thus, fungus-exposed aphids differed from the individuals from the control group mainly in the conidia covering their surface. Encountering the experimental aphids, *L. fuliginosus* milkers had to assess the potential hazard level of the ‘newcomers’. To remove excess liquid after the treatment, experimental aphids were placed on a clean filter paper for about 4–5 s. Right after that, the aphid was placed on a plant relatively close to the aphid colony being observed (at a distance of about 10–15 cm), when no milkers were around. To avoid damaging experimental aphids, fine paintbrushes were used to transfer insects. After being placed on a plant, experimental aphids tended to move down, join other aphids in a colony and not change their location up to the end of a test. In addition to direct observation, the behaviour of insects was recorded on video in order to examine some points in detail if needed. In isolated cases when an experimental aphid was confused with other individuals on the plant, the test was stopped and excluded from consideration.

Tests were carried out for separate ant-tended aphid colonies. In each case, experimental aphids (one conidia-contaminated and one uncontaminated individual) were introduced to the model aphid colony in a random order, with about 30–60 min between tests. Each experimental aphid was used only once. If a contaminated experimental aphid was not rejected by ants and stayed on the plant, it was removed after the test to avoid possible contamination of other aphids in the colony. At the beginning of each test, the number of ants and aphids presented in the aphid colony were noted. Behaviour of ants and experimental aphids was recorded until the treated aphid was removed from the plant, but not for longer than 5 min after its first encounter with milkers. The time an aphid spent in the aphid colony after its first encounter with milkers (‘survival time’) was recorded. Because the experiments were carried out in nature and experimental aphids were treated just before the testing, it was almost impossible to record data blindly. Nevertheless, observer bias was minimized by the choice of quite unequivocal behavioural parameters of registering: (1) removal ant behaviour (aphid was removed or not); (2) aphid removal technique used by milkers (throwing aphid away or carrying it down the tree trunk; placing it at some distance from the aphid colony or carrying it to the nest); (3) aggressive ant reactions (‘hit-and-run attacks’, biting, ‘death grip’—prolonged bite with a bent abdomen, as if an ant was spraying acid on the aphids); (4) non-aggressive ant reactions (neutral reaction, antennation, soliciting honeydew, grooming); (5) the secretion of honeydew by the experimental aphid. In total, 86 observations (tests) in 43 aphid colonies were recorded in three ant colonies of *L. fuliginosus*. Descriptive statistics for the aphid colonies investigated are presented in Table S1.

### 2.3. Data Analysis

To analyse removal of experimental aphids, a Cox proportional-hazard regression model was used, with treatment (fungus-contaminated or not), the number of aphid milkers (N_AM_) and aphid colony size (N_APH_) used as independent variables. To compare pairs of survival curves (i.e., proportion of experimental aphids which were not removed by milkers, as a function of time) Kaplan–Meier survival analysis was used with Gehan’s Wilcoxon Test. 

To exclude the different influence of the ant colony and the presentation sequence of experimental aphids (order) on aphid removal, the main effects, treatment (fungus-exposed or not), colony (*n* = 3), order (1 or 2), as well as interaction of treatment and ant colony (treatment * colony), and treatment and order (treatment * order) were analysed using Generalized Linear Models (GLMs), with binomial distribution.

To test the impact of fungal contamination on the displayed aggressive reactions by *Lasius fuliginosus* milkers when encountering experimental aphids—*n* = 117 (ant demonstrated at least one of the aggressive reactions mentioned above or not), excluding the differing influence of some other factors—the main effects of treatment (fungus-exposed or not), colony (*n* = 3), the number of aphid milkers (N_AM_) and aphid colony size (N_APH_), as well as interaction of treatment and ant colony (treatment * colony) were analysed using Generalized Linear Models (GLMs), with binomial distribution. 

To analyse the responses of milkers towards conidia-contaminated and uncontaminated aphids after first encounter—*n* = 117 (removing experimental aphid; tending aphid: soliciting honeydew; no reaction: ants pay no attention to tested aphid)—a GLM with multinomial distribution was used. The percentages of ant responses towards experimental aphids were compared with Fischer’s exact two-tailed test.

To test the hypotheses, an alpha level of 0.05 was used throughout. The data were analysed with STATISTICA v.8.0.725 (StatSoft, Tulsa, OK, USA).

## 3. Results

**Figure 2:**
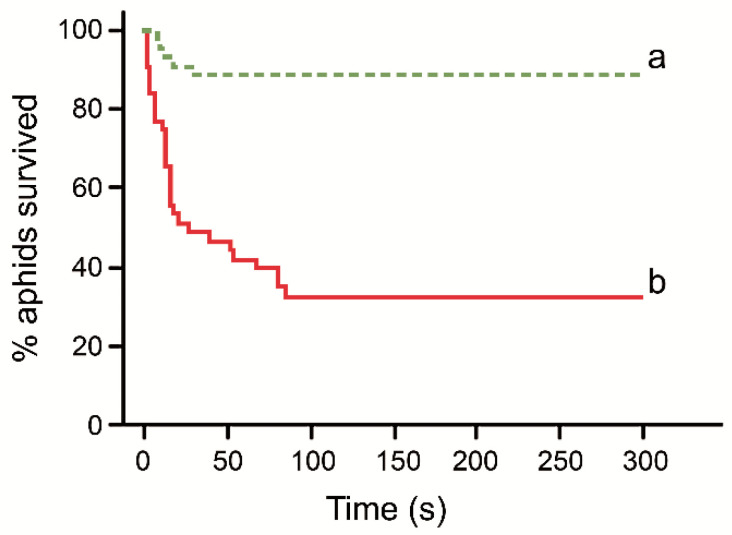
The percentage of experimental *Chaitophorus populeti* aphids—contaminated with *Beauveria bassiana* (red solid line) or non-contaminated control aphids (green dotted line)—non-removed by aphid milkers of *Lasius fuliginosus* (‘survived’) as a function of time. Different letters at the end of the curves indicate significantly different removal in pair-wise comparisons using Kaplan–Meier survival analysis (Gehan’s Wilcoxon = −5.06, *p* < 0.0001).

Aphid removal by *L. fuliginosus* aphid milkers was highly associated with the fungal contamination of aphids (*χ*^2^ = 30.80, d.f. = 3, *p* < 0.0001): conidia-contaminated aphids were removed faster than experimental aphids from the control group (Table S2). Experimental aphids covered with conidia had a hazard ratio of removal 8.9 times higher than uncontaminated aphids (Wald = 20.08, d.f. = 1, *p* < 0.0001; Figure 2). Removal of experimental aphids did not depend on the number of aphid milkers (Wald = 0.65, d.f. = 1, *p* = 0.42) or the aphid colony size (Wald = 1.56, d.f. = 1, *p* = 0.21). The ant colony and the presentation sequence of experimental aphids (order) had no significant effects on the proportion of aphids removed from the plant by *L. fuliginosus* aphid milkers (Table 1, Table S3).

**Figure 3:**
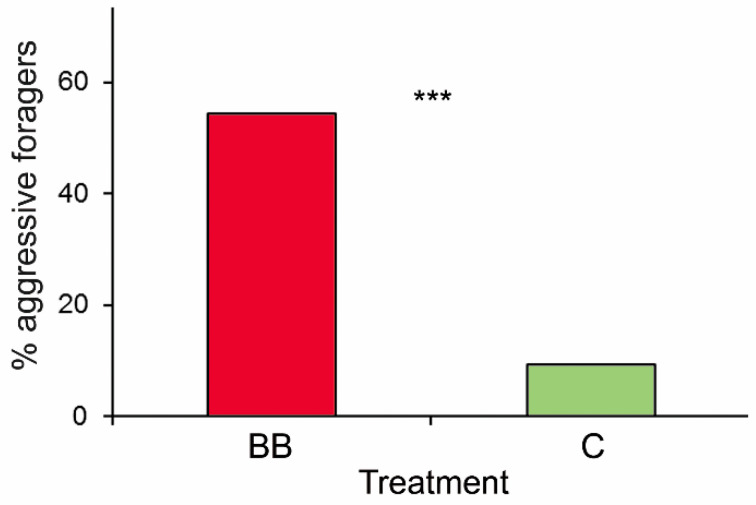
The percentage of *Lasius fuliginosus* aphid milkers demonstrating aggressive reactions during their first encounter with *Chaitophorus populeti* aphids contaminated with *Beauveria bassiana* (BB) or uncontaminated control aphids (C). Generalized Linear Model (GLM), with binomial distribution: *** *p* < 0.0001.

The number of milkers that interacted with experimental aphids per trial varied from 1 to 4 in tests with both fungus contaminated and control aphids (mean ± SE: 1.39 ± 0.11 and 1.3 ± 0.09, respectively) and totalled 117 milkers (60 and 57 milkers, respectively). The proportion of foragers acting aggressively during their first encounter with experimental aphids was much higher in tests with conidia-contaminated aphids than in the control group (Table 1, Figure 3). The number of milkers that demonstrated aggressive and non-aggressive reactions during their first encounter with experimental aphids is presented in Table S4. The ant colony, the number of milkers, or the aphid colony size had no significant effects on the behaviour of *L. fuliginosus* milkers towards experimental aphids (Table 1). 

**Figure 4:**
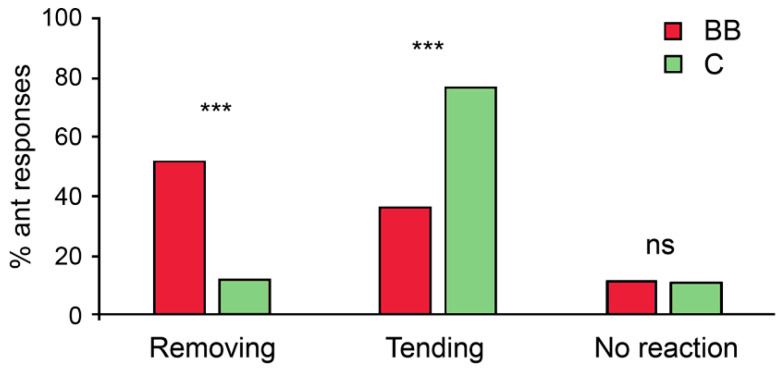
The percentage of different responses of *Lasius fuliginosus* aphid milkers towards *Chaitophorus populeti* aphids contaminated with *Beauveria bassiana* (BB) or uncontaminated control aphids (C) immediately after their first encounter. Fischer’s exact two-tailed test: *** *p* < 0.0001, ns—*p* > 0.05.

The behaviour of milkers immediately after encountering an experimental aphid was also significantly affected by the type of treatment (χ2 = 23.85, d.f. = 2, *p* < 0.0001). Conidia-contaminated aphids were much more often removed from the plant and were less likely to be tended by *Lasius fuliginosus* milkers than uncontaminated aphids (Figure 4). The number of milkers that demonstrated different responses towards experimental aphids immediately after their first encounter is presented in Table S5. In the tested aphid colonies, the number of milkers demonstrating aggressive reactions towards conidia-contaminated aphids during their first encounter was not high; in trials where 2–4 milkers interacted with the experimental aphids, no more than one aggressive worker was observed. Besides, subsequently, some of these aggressive milkers did not remove experimental aphids. As a result, immediately after encountering, the percentage of *L. fuliginosus* foragers tending fungus contaminated aphids was quite high (Figure 4) and did not differ significantly from the percentage of milkers removing aphids covered with conidia (Fischer’s exact two-tailed test, *p* = 0.1411).

Only the most aggressive honeydew foragers of *L. fuliginosus* immediately detected and removed fungus-exposed aphids from the plant. These ants grabbed conidia-contaminated aphids with their mandibles just after their first encounter and tried to tear them away from the plant (within 1–2 s). After that, milkers bent their abdomens round as if they were spraying acid onto the grabbed aphids (about 1–5 s) and finally carried the attacked and apparently killed aphid down the plant trunk to the ground (Figure 1). Meanwhile, the aphid usually excreted a droplet of honeydew, which was actively licked by other non-aggressive or less aggressive milkers, which tried to stroke the experimental aphid with their antennae, grooming it and soliciting honeydew.

The removal behaviour of *Lasius fuliginosus* was limited to only one technique, which was used by milkers to dispose of some experimental aphids. The grabbed unsuitable individuals were carried down the trunk. None of experimental aphids were flung away from the plant. While selected uncontaminated aphids were transported to the nest, conidia-contaminated aphids were usually placed into small holes in the ground or under dry leaves some distance (15–30 cm) away from the base of the experimental tree. None of the removed fungus-exposed aphids were carried to the ant nest. After getting rid of the fungus contaminated aphid, workers groomed themselves for about 2–3 min and only then did they return to their aphid colony.

## 4. Discussion

Although the results of the first study of quarantining behaviour in ants towards fungus infected aphids were published ten years ago [33], still very little is known about the range of ants which can actively use this behavioural pattern. Among seven studied ant species, only ants of the genus *Formica* were found to be able to quickly detect fungus contaminated aphids and dispose of them [33,34,35]. Milkers of *Myrmica rubra* L. and *M. scabrinodis* Nylander, as well as *Lasius niger*, the only ant of the genus *Lasius* studied so far, tended to demonstrate non-aggressive reactions towards aphids covered with conidia [34,35]. The study of the reaction of *L. fuliginosus* towards fungus-contaminated aphids has shown that quarantining behaviour is not limited to the ants of the genus *Formica*. The experimental investigation of the reaction of *Lasius fuliginosus* milkers towards conidia-contaminated aphids carried out in nature indicated that ants of the genus *Lasius* can also make use of this antifungal mechanism to prevent infection transmission. As was expected, some aphid milkers of *L. fuliginosus* quickly detected aphids contaminated with *Beauveria bassiana* conidia and carried them far away from the aphid colony. Before removal, most of the attacked fungus-exposed aphids were apparently disinfected with a poisonous secretion of the ants (Figure 1C) and killed. However, whether aphids were really sprayed with poison and killed or not was not checked during the research. Removed conidia-contaminated aphids were carried down the plant and placed into small holes in the ground or under dry leaves some distance away from the base of the experimental trees. Neither the number of milkers nor the size of the examined aphid colonies affected the removal behaviour of *L. fuliginosus* towards the experimental aphids. The percentage of conidia-covered aphids removed from the plant was found to be much higher than in the control (67.4% vs. 11.6%). These results appear to be similar to those obtained earlier for *Formica* s. str. ants: *F. polyctena*—75.9% vs. 16.7%, *F. rufa*—92.6% vs. 9.4%, *F. pratensis*—87.0% vs. 26.9%, correspondingly [34]. Despite both of these experimental investigations being carried out with the same methodology using fungal pathogen *B. bassiana*, the extant data of aphid removal is difficult to compare because it is impossible to exclude the impact of some important factors (e.g., aphid species, year, etc.). Nevertheless, unlike *Lasius niger*, both *Formica* s. str. ants and *L. fuliginosus* quite quickly detected and removed most of the fungus-contaminated aphids. It is worth noting, however, that some milkers, of both *Formica* s. str. and *L. fuliginosus*, were quite tolerant towards fungus contaminated aphids, demonstrating a neutral reaction or actively tapping them with their antennae, grooming them, and licking excreted honeydew. Removal behaviour was most typical of highly aggressive foragers that immediately attacked fungus contaminated aphids, paying no attention to the honeydew droplets excreted by these aphids. However, in *Formica* s. str., this was a characteristic behaviour of the ‘guards’ protecting the aphids [34], which are constantly present on the plant with their aphid symbionts along with ‘shepherds’ collecting honeydew [45]. As for *L. fuliginosus*, unspecialised milkers of this species, independent of their aggressiveness, have to leave the plant from time to time to transport collected honeydew to the nest [45]. When ‘aggressive’ milkers left the plant to carry honeydew to the nest, the effectiveness of aphid protection could be decreased. So, in four cases, milkers had been demonstrating non-aggressive reactions towards fungus contaminated aphid for several minutes after the first encounter, until one of foragers, just returned from the nest, immediately detected and removed a fungus-exposed aphid. Some minor differences concern aphid removal technique used by milkers as well. Ants of *Formica* s. str. are known to use various techniques to dispose of detected and grabbed aphids, including the predominate ‘throwing aphid away from the plant’ (about 70%), ‘carrying aphid down the trunk’ (about 25%) and, the most rarely observed, ‘placing more than 15 cm away from the aphid colony’ [34]. Unlike *Formica* s. str., milkers of *Lasius fuliginosus* always carried attacked conidia-contaminated aphids to the base of the host-plant and left them some distance from the nearest foraging trail, placing these aphids into small holes in the ground or under dry leaves. To assess the effectiveness of these techniques of aphid removal in preventing infection transmission, additional detailed investigation is required.

*Lasius fuliginosus* is known to be able to assess the ‘quality’ of their symbionts, eliminate unsuitable aphid individuals and transport them to the nest as protein food [46]. However, none of the fungus contaminated aphids removed from the plant were observed being transported to the nest while testing was in progress. While foragers of some ants (e.g., *Myrmica* spp.) can collect prey covered with conidia [54], the ability of *L. fuliginosus* to differentiate fungus contaminated aphids among unsuitable individuals and avoid carrying them to the nest minimizes sanitary risks for the colonies of this species. Generally, the quarantining behaviour of *L. fuliginosus* and *Formica* s. str. seems to be quite similar, removing most of the fungus contaminated aphids from the plant enables these ants to reduce the risk of infection transmission among both symbiont partners. This is the first evidence of active usage of quarantining behaviour to prevent transmission of infection disease in *Lasius* ants. Unlike *L. fuliginosus*, *L. niger* tend to demonstrate non-aggressive reactions towards aphids covered with *B. bassiana* conidia; the percentage of fungus contaminated aphids removed from the plant was found to be low and did not differ significantly from the control, 18.2% vs. 9.1%, respectively [34]. At the same time, *L. niger* is known to be able to differentiate between dead uninfected and fungus-infected aphid cadavers [55]. Unlike uninfected corpses, sporulating cadavers of aphids after infection by the fungal entomopathogen *Lecanicillium longisporum* (Zimmerman) Zare and Gams were removed much faster, and they were most often moved, not to the ant nest, but to the corner of the box containing the ant nest or into a specific place where the dead ants were disposed of [55]. The reasons *L. niger* milkers ignore living aphids externally contaminated with conidia of *B. bassiana* might be explained by the usage of some specific methods of fighting disease. For instance, *L. niger* is known to contain quite a high number of cytochrome P450 genes that can enable this species to neutralize various toxins, including mycotoxins, and thereby decrease the risk of infection, thereby encountering low levels of conidia [56]. Besides, the different reactions of *L. niger* and *L. fuliginosus* towards conidia-contaminated aphids could potentially be explained by the significant differences both in the colony size and the social and territorial organization of these ants. While *Lasius fuliginosus* is similar to *Formica* s. str. ants due to its large colonies (10^5^–10^6^ individuals) and vast protected territories, *L. niger* lives in smaller colonies (10^2^–10^4^ individuals) with non-protected or partially protected territory [2,41]. The larger ant colonies need much more carbohydrates, which requires more effective exploitation of aphids, including active protection of aphid colonies because of the increase in the number of milkers tending aphids [45]. The more milkers there are and the more contact there is leads to a greater risk of milkers being a disease vector that can transmit pathogens both to their nestmates and other aphids. So, with an increase of colony size, ants seem to need additional mechanisms to keep their carbohydrate resources stable and prevent infection transmission among both their nestmates and trophobionts. This is consistent with the fact that the efficiency and the type of hygienic behaviours of ants are determined by colony size and the sanitary threat [20,57,58]. Nevertheless, to check this supposition, additional thorough investigation is required.

## 5. Conclusions

In general, active usage of quarantining behaviour towards fungus contaminated aphids appears to be not limited to the genus *Formica*. It was found to be typical of at least one species of *Lasius*, *L. fuliginosus*, living in large colonies with complex territorial organization similar to ants of *Formica* s. str. Some milkers of *L. fuliginosus*, those that seem to be the most aggressive, can quickly detect and remove aphids covered with conidia of generalist fungi *Beauveria bassiana* from a plant, as honeydew foragers of *Formica* s. str. tend to do. The attacked fungus contaminated aphids were carried down the plant and placed into small holes in the ground or under dry leaves some distance away from the base of the experimental trees. In addition, most were apparently disinfected using poison secretion. Th response of aphid milkers of *L. fuliginosus* and *Formica* s. str. to living aphids externally contaminated with conidia of *B. bassiana* is quite similar. Removal of most fungus-exposed aphids from the plant enables these ants to reduce the risk of infection transmission among both their nestmates and symbiont partners.

## Figures and Tables

**Table 1:** Statistical analysis of the behaviour of *Lasius fuliginosus* milkers towards *Chaitophorus populeti* experimental aphids contaminated with *Beauveria bassiana* (BB) or not (C). With a Generalized Linear Model (GLM), with binomial distribution, the association of aphid removal (aphid was removed or not) with treatment, ant colony (*n* = 3), the presentation sequence of experimental aphids (order: 1 or 2) and the association of aggressive behaviour (ant encountering experimental aphid displayed aggressive reactions or not) with treatment, ant colony, the number of aphid milkers (N_AM_) and aphid colony size (N_APH_) were analysed. The sign (×) indicates the interaction of two factors influencing ant behaviour.

Response Variable	Explanatory Variables	d.f.	Wald Stat.	*p*-Value
Removal of experimental aphids	Treatment	1	26.22	<0.0001
	Ant colony	2	0.55	0.76
	Order	1	0.01	0.94
	Treatment × Ant colony	2	0.43	0.81
	Treatment × Order	1	0.73	0.39
The percentage of ants acting aggressively towards experimental aphids	Treatment	1	26.04	<0.0001
	N_AM_	1	0.60	0.44
	N_APH_	1	1.11	0.29
	Ant colony	2	0.37	0.83
	Treatment × Ant colony	2	0.52	0.77

## Data Availability

The data supporting reported results are available in the Supplementary Materials of this article.

## Supplementary Materials

The following are available online at https://www.mdpi.com/2075-4450/12/2/99/s1: Table S1: Descriptive statistics for aphid colonies investigated: the number of aphids (N_APH_) and aphid milkers (N_AM_); Table S2: The ‘Survival’ time (s) of Chaitophorus populeti aphids contaminated with Beauveria bassiana (BB) and individuals from the control group (C) in aphid colonies attended by Lasius fuliginosus; Table S3: The number of experimental aphids contaminated with Beauveria bassiana (BB) or uncontaminated (C) that were removed or not removed by Lasius fuliginosus milkers during tests; Table S4: The number of Lasius fuliginosus aphid milkers demonstrating aggressive and non-aggressive reactions during their first encounter with experimental aphids contaminated with Beauveria bassiana (BB) or not (C); Table S5: The number of Lasius fuliginosus milkers demonstrating different responses towards experimental aphids contaminated with Beauveria bassiana (BB) or not (C) immediately after their first encounter.
