# Supplementary material for: Preventing Transmission of Lethal Disease: Removal Behaviour of Lasius fuliginosus (Hymenoptera: Formicidae) Towards Fungus Contaminated Aphids
Source: Insects. 2021 Jan 24;12(2):99. doi: 10.3390/insects12020099 (PMC7911655; doi:10.3390/insects12020099)
Supplement: Supplementary file 1 [file insects-12-00099-s001.pdf]

Preventing transmission of lethal disease: Removal behaviour of *Lasius fuliginosus* (Hymenoptera: Formicidae) towards fungus contaminated aphids

Supplementary material

Tatiana Novgorodova *

Table S1. Descriptive statistics for aphid colonies investigated: the number of aphids (N_{APH}) and aphid milkers (N_{AM}).

Ant colony	Number of aphid colonies	Parameter	Median	Range (Min–Max)	Quartile range [25%; 75%]
Lful_1	15	N_{APH}	11.0	10.0 (6.0–16.0)	4.0 [9.0; 13.0]
		N_{AM}	2.0	3.0 (1.0–4.0)	1.0 [1.0; 2.0]
Lful_2	14	N_{APH}	13.5	20.0 (9.0–29.0)	7.0 [12.0; 19.0]
		N_{AM}	3.0	4.0 (1.0–5.0)	1.0 [2.0; 3.0]
Lful_3	14	N_{APH}	12.5	14.0 (7.0–21.0)	5.0 [9.0; 14.0]
		N_{AM}	2.0	4.0 (1.0–5.0)	1.0 [2.0; 3.0]

Table S2. 'Survival' time (s) of *Chaitophorus populeti* aphids contaminated with *Beauveria bassiana* (BB) and individuals from the control group (C) in aphid colonies attended by *Lasius fuliginosus*.

Effect	Level of factor	Level of factor	N	Residence ('survival' time)				
				Mean	Std.Dev.	Std.Err.	-95%CI	+95%CI
Total			86	190.17	137.47	14.82	160.70	219.65
Ant colony	1		30	197.77	137.37	25.08	146.47	249.06
	2		28	191.86	137.80	26.04	138.42	245.29
	3		28	180.36	141.66	26.77	125.43	235.29
Treatment	BB		43	113.49	132.85	20.26	72.60	154.37
	C		43	266.86	92.48	14.10	238.40	295.32
Treatment * Ant colony	1	BB	15	114.40	137.32	35.45	38.36	190.44
	1	C	15	281.13	73.07	18.87	240.67	321.60
	2	BB	14	123.86	138.02	36.89	44.17	203.55
	2	C	14	259.86	102.12	27.29	200.90	318.82
	3	BB	14	102.14	131.94	35.26	25.96	178.33
	3	C	14	258.57	105.31	28.15	197.77	319.38

Table S3. The number of experimental aphids contaminated with *Beauveria bassiana* (BB) or uncontaminated (C) that were removed or not removed by *Lasius fuliginosus* milkers during tests.

Effect	Level of factor	Level of factor	N	Total count	Non-removed	Removed
Total			86	86	52	34
Treatment	BB		43	43	14	29
	C		43	43	38	5
Ant colony	1		30	30	19	11
	2		28	28	17	11
	3		28	28	16	12
Order	1		43	43	27	16
	2		43	43	25	18
Treatment * Ant colony	BB	1	15	15	5	10
	BB	2	14	14	5	9
	BB	3	14	14	4	10
	C	1	15	15	14	1
	C	2	14	14	12	2
	C	3	14	14	12	2
Treatment * Order	BB	1	21	21	8	13
	BB	2	22	22	6	16
	C	1	22	22	19	3
	C	2	21	21	19	2

Table S4. The number of *Lasius fuliginosus* aphid milkers demonstrating aggressive and non-aggressive reactions during their first encounter with experimental aphids contaminated with *Beauveria bassiana* (BB) or not (C).

Effect	Level of factor	Level of factor	N	Total count	Non-aggressive	Aggressive
Total			117	117	79	38
Ant colony	1		33	33	21	12
	2		48	48	35	13
	3		36	36	23	13
Treatment	BB		60	60	27	33
	C		57	57	52	5
Treatment * Ant colony	1	BB	18	18	7	11
	1	C	15	15	14	1
	2	BB	23	23	12	11
	2	C	25	25	23	2
	3	BB	19	19	8	11
	3	C	17	17	15	2

Table S5. The number of *Lasius fuliginosus* milkers demonstrating different responses towards experimental aphids contaminated with *Beauveria bassiana* (BB) or not (C) immediately after their first encounter.

Treatment	Total number of ants	Ant responses		
		Removing	Tending	No reaction
BB	60	31	22	7
C	57	7	44	6
